# Increasing emergency department admissions for chemsex-related intoxications in Barcelona, Spain, among people living with HIV: an observational study from 2018 to 2020

**DOI:** 10.1186/s12889-022-12763-3

**Published:** 2022-02-18

**Authors:** Gabriel Vallecillo, Alejandra Losada, Alexy Inciarte, Chen Jiwei, Albert Monterde, Emilio Salgado, Adriana Laura Doi, Esther Monclus, Rafael Perelló

**Affiliations:** 1 Drug addiction Unit, Hospital del Mar, Parc de Salut Mar Consortium, Barcelona, Spain; 2 Emergency Department, Hospital Clínic, C. de Villarroel, 170, 08036 Barcelona, Spain; 3 Infectious Diseases Department, Hospital Clínic, Barcelona, Spain

**Keywords:** PLHIV, Chemsex, Intoxications, Drugs, Mental illness

## Abstract

**Background:**

Chemsex is a novel phenomenon referring to the use of drugs, including crystal methamphetamine, gammahydroxybutyric acid (GHB)/gamma-butyrolactone (GABA) and mephedrone, to facilitate, enhance, and prolong the sexual experience in men who have sex with men in large cities internationally. There is a growing concern about chemsex and fatal cases among people living with HIV on antiretroviral therapy. This study aimed to describe the clinical characteristics of chemsex-related intoxications.

**Material and methods:**

An observational study was conducted in people living with HIV who were admitted for chemsex-related intoxications in an emergency department of a teaching hospital in Barcelona, Spain, from 2018 to 2020. Severe acute intoxications were defined according to the Poisoning Severity Score.

**Results:**

One hundred and fifteen male patients with a median age of 35.6±7 years were included in the study:15 (13.1%) in 2018, 32 (27.8%) in 2019 and 68 (59.1%) in 2020. All patients had stable housing, 107(93.0%) were Spanish citizen and 32 (27.8%) had mental health disorders. Median CD4 lymphocyte count was 624 (500–765) cells/mm^3^ and 99 (86.1%) had HIV-1 RNA suppression.

Poly-drug use was observed in 51(44.3%) cases and methamphetamine in 75(65,2%) and gammahydroxibutiric acid in 68 (59.1%) were the main drugs used.

Potential drug interactions due to the inhibition of cytochrome P450 by antiviral therapy was determined in 36 (31.3%) patients. Severe intoxications cases affecting neurologic and respiratory systems were diagnosed in 12 (10.4%) patients and no patient died. CD4 cell counts ≤500 cells (O.R.:4.2; C.I.95%:1.2–14.6) and mental health disorders (O.R.: 2.9; C. I 95%: 0.8–9.9) were associated with severe acute drug intoxications in the bivariate analyses.

**Conclusions:**

Chemsex-related intoxications are an increasing clinical problem in people living with HIV. Chemsex should be routinely screened and addressed in clinical practice, particularly for people with mental illness and low CD4 cell counts, who are at higher risk for severe intoxications.

## Background

Chemsex is defined as the intentional use of psychoactive or non-psychoactive drugs before or during sex to facilitate, prolong, and/or intensify sexual experience. Drugs can also be injected, in a behaviour called ‘slamming’ or ‘slamsex’ [1, 2]. It is practiced mainly by men who have sex with men (MSM) [1, 2], and the most common used drugs are crystal methamphetamine, gammahydroxybutyric acid (GHB)/gamma-butyrolactone (GABA) and mephedrone [1, 2].

The prevalence of chemsex ranges from 3 to 29% according to the measurement and recruitment methods used [3–7]. However, it has been found that people living with HIV (PLHIV) may practice chemsex more frequently than the ones who have HIV negative test results or with an unknown status [3–7].

Nevertheless, chemsex drugs use is associated with serious consequences on the health of PLHIV [5]. First, drug use may interfere with daily routine and consequently with adherence to antiretroviral therapy increasing the risk of virologic failure [8–10]. Second, drugs use is associated to sex without condoms and high-risk sexual behaviours [11] facilitating transmission of hepatitis C virus and other sexual transmitted infections [11–14]. Third, substance-using sexual and gender minorities engaging in chemsex are at increased risk of sexual violence [15–17]. Finally, chemsex drugs have the potential to interact with antiretroviral drugs because they are metabolized, at least in part, by the CYP450 system, which could lead to affect antiviral plasma concentrations or increase recreational drugs toxic effects [18, 19] resulting in acute intoxications [20–25]. Specifically, boosting agents ritonavir and cobicistat have moderate inhibitory effects on the metabolism of methamphetamine and mephedrone and low with GHB. It is important to note that the extent of the interaction varies according to the route of administration, as these drugs can be administrated by different ways, including oral, nasal, intravenous and rectal [18, 19].

However, clinical data available come from case series reported due to the ethical impossibility of conducting clinical trials under these clinical circumstances.

Therefore, it is necessary to describe the incidence and clinical factors related to acute chemsex intoxications with the purpose of identifying patients at higher risk, evaluating the impact of drug-drug interactions and stablishing preventive measures.

The aims of this study were to describe the severity and related factors of acute chemsex intoxications in PLHIV admitted to an emergency department.

## Methods

This retrospective study was conducted in the emergency department of a teaching hospital of Barcelona (Spain) from January, 2018 to December, 2020.

All PLHIV who were admitted for acute drug intoxication associated to chemsex in the emergency department during the study period were included in the study. For the purpose of the study, demographic and clinical characteristics, including mental health disorders, which were diagnosed according to the DSM-V previously to the admission, were extracted from the patients’ medical records and categorized to maintain anonymity where necessary. Chemsex was defined as the intentional sex under the influence of one or more of the next drugs: crystal methamphetamine, gammahydroxybutyric acid (GHB)/gamma-butyrolactone (GABA) or mephedrone [1]. Poly-drug use was considered as the use of two or more chemsex drugs. Acute drug intoxication was defined as a transient clinical condition following the administration of chemsex drugs, resulting in disturbances in level of consciousness, cognition, perception, affect or behavior, or other psychophysiological functions and responses [26]. Severe acute intoxications were defined according to the Poisoning Severity Score [27]. Chemsex drug detection tests were performed by immunoassay (DRI®; Abbott Diagnostics, Texas, USA), and confirmation and detection of interference by gas chromatography and mass spectrometry (GC-MS) (Agilent 5975/68901, Santa Clara, CA, USA). A specific method was used for the detection of GHB [28]. Potential interactions of antiretrovirals with chemsex drugs were based on in vitro effects of antiretrovirals on the cytochrome P450 and categorized as induction, for efavirenz, nevirapine or etravirine, but not for ritonavir-boosted protease inhibitors; inhibition, for ritonavir or cobicistat boosted protease inhibitors and elvitegravir regardless of other drugs included in the regimen; or neutral for other regimens excluding ritonavir/cobicistat or efavirenz/nevirapine/etravirine but including rilpivirine, raltegravir, dolutegravir, or maraviroc based regimens [18, 19].

The primary end point of the study was the incidence of severe acute chemsex drug intoxications, defined as the number of severe acute intoxications/number of total intoxications quotient, and related factors.

Descriptive statistics were expressed as mean, standard deviation, median and range for continuous variables and absolute frequencies and percentages for categorical variables. The chi-square test was used to compare categorical variables and Mann-Whitney U test for continuous variables that did not follow a normal distribution. Bivariate analyses was used to assess clinical factors associated to severe acute intoxications. A *p* value < 0.1 was considered significant. Analyses were made using SPSS software, version 17.0.0 (Chicago, Illinois).

The study complied with the ethical statements in the Declaration of Helsinky (64th General Assembly, Fortaleza, Brazil, October 2013) and was approved by the local Ethics Committee (Ethics Committee for Drug Research, Clinic Hospital Clinic, Barcelona, Spain, HCB-2021-004). The ethics committee waived the need of informed consent due to the retrospective design of the study.

## Results

During the study period 119 patients were admitted to the emergency department for an acute chemsex drug intoxication, of whom 115 were PLHIV: 15 (13.1%) in 2018, 32 (27.8%) in 2019 and 68 (59.1%) in 2020.

Clinical characteristics of the patients included in the study are shown in Table 1. Most of the individuals were Spanish citizen men who have sex with men and all patients had stable housing. Main mental health disorders were: personality disorders in 19 patients, anxiety and mood in 11 and bipolar in 2. Immunoglobulin G antibodies against hepatitis C virus antigens were positive in 13 patients and hepatitis C virus RNA was positive in 2 patients.

**Table 1:** Clinical characteristics of the 115 people living with HIV with acute intoxication associated to chemsex

Characteristics	Total	Severe acute intoxication	Non-severe acute intoxication	*p*
n	115	12 (10.4%)	103 (89.6%)	
Age ^1^	35.6±7	35.3±8	35.4±7	0.94
MSM	111 (96.5%)	12 (100%)	99 (89.5%)	0.64
Bisexual men	4 (3.5%)	0 (0.0%)	4 (10.5%)	
Spanish citizen	107 (93.0%)	11 (91.7%)	96 (93.2%)	0.84
Stable housing	115 (100%)	12 (100%)	103 (100%)	1
Mental health disorder	32 (27.8%)	7 (58.3%)	25 (24.3%)	0.02
IgG antibodies hepatitis C	13 (11.3%)	1 (9.1%)	12 (13.1%)	0.89
CD4 lymphocyte				
total ^2^	624 (500–765)	518 (395–639)	630 (512–800)	0.04
≥500 cells/mm3	89 (77.3%)	6 (50%)	83 (80.6%)	0.03
VIH-1 RNA < 50 copies/mL	99 (86.1%)	9 (75.0%)	90 (87.4%)	0.22
IGI	78 (67.8%)	9 (75%)	69 (67.0%)	
NNRTI	22 (19.1%)	1 (8.3%)	21 (20.4%)	0.53
PI	15 (13.0%)	2 (16.7%)	13 (12.6%)	
Inhibitor drug-drug interactions	36 (31.3%)	4 (33.3%)	32 (31.1%)	0.55
Poly-drug use	51 (44.3%)	5 (41.6%)	46 (44.6%)	0.84

Data are presented as No. (%) unless otherwise indicated.1: Data presented as mean ± standard deviation. 2: Data presented as median and interquartile range

*Abbreviations*: *MSM* Men who have sex with men, *IgG* Immunoglobulin, *mL* Mililiter, *RNA* Ribonucleic acid, *IGI* Integrase inhibitors, *NNRTI* Non-nucleoside reverse transcriptase inhibitor, *PI* Protease inhibitor

HIV-1 RNA suppression was observed in 99(86.1%) patients with a median CD4 cell count of 624 cells/mm3. Integrase inhibitors were the main antiviral group used for HIV treatment.

Poly-drug use was observed in 51(44.3%) patients and the main drugs detected were: methamphetamine in 75 (65.2%) cases, GHB in 68 (59.1%), cocaine in 40 (34.7%), ketamine in 12(10.4%), amyl nitrite in 12 (10.4%) and mephedrone in 7 (6.1%) and alcohol in 19(16.5%).

One third of the antiretroviral-based regimens had a potential inhibition drug-drug interaction: elvitegravir/cobicistat with methamphetamine in 13 cases, with mephedrone in 4, with GHB in 3, with cocaine in 2 and ketamine in 1; and darunavir/cobicistat with methamphetamine in 10, with mephedrone in 2 and with GHB in 1.

Severe acute chemsex drug intoxications were diagnosed in 12(10.4%) patients, presenting as neurological alterations in the form of coma with respiratory failure. No patient died. The bivariate analysis of factors associated to severe intoxications is shown in Table 2. CD4 cell count ≤500 cells and mental health disorder were associated to severe acute drug intoxications in the bivariate analysis. No specific chemsex drugs were associated to the severity of acute intoxications.

**Table 2:** Bivariate analysis of clinical factors associated with acute severe chemsex intoxications

Characteristics	O.R.	*p*
Age	1.1 (0.9–1.11)	0.61
MSM		
Bisexual men	1.1 (0.1–22.2)	0.93
Spanish citizen	0.8 (0.1–7.1)	0.84
Stable housing	0.12 (0.1–6.3)	0.29
Mental health disorder	2.9 (0.8–9.9)	0.08
IgG antibodies hepatitis C	0.7 (0.1–5.8)	0.77
CD4 lymphocyte		
< 500 cells/mm3	4.2 (1.2–14.6)	0.02
VIH-1 RNA < 50 copies/mL	0.4 (0.1–1.8)	0.25
IGI	1	
NNRTI	0.8 (0.1–13.7)	0.90
PI	3.4 (0.3–35.9)	0.31
Inhibitor drug-drug interactions	1.1 (0.3–3.9)	0.87
Poly-drug use	0.9 (0.3–2.9)	0.62

*Abbreviations*: *O.R.* Odds ratio, *MSM* Men who have sex with men, *IgG* Immunoglobulin, *mL* Mililiter, *RNA* Ribonucleic acid, *IGI*, Integrase inhibitors, *NNRTI* Non-nucleoside reverse transcriptase inhibitor, *PI* Protease inhibitor

## Discussion

The results of this observational study showed that the number of acute chemsex drug intoxications increased over the study period and that the severity of the intoxications was higher in people with low CD4 cell count and mental health disorders.

Immune impairment was the main factor associated with the severity of acute intoxications in this study. In this sense, cocaine, alcohol, cannabinoids and ketamine, which are not primarily considered to be immunosuppressive agents, have been described to modulate the humoral and cellular immune response in humans or animals [29, 30]. Otherwise, the low CD4 count could only be indicative of a worse overall clinical situation, and therefore point to those individuals more susceptible to the effects of chemsex drugs, similar to the adverse event of antiretroviral therapy, which progressively increase with decline in CD4 cell count [31]. Finally, this association could only reflect poor adherence to antiretroviral therapy and lower immune recovery. However the observational design of the study does not allow to contrast these hypothesis.

The other factor related to the severity of intoxications was the mental illness. This association is relevant as its prevalence in PLHIV are higher than the general population [32–35] as a result of the bidirectional relationship between both conditions [36, 37]. Moreover, PLHIV with mental health disorders often have substance use disorders [38, 39], and the co-occurrence of these conditions impact negatively on the clinical outcomes and the patient’s quality of life [34–36]. In this sense, the severity of acute drug intoxications observed in patients with mental health disorders in this study could be explained by other drug-drug interactions between chemsex drugs and psychiatric drugs used for the treatment of mental health disorders [40]. Nevertheless, the association of mental disorders with the severity of intoxications highlights the importance of checking and addressing routinely the use of drugs in PLHIV, particularly in whose with mental health disorders [41].

It is also important to comment that drug-drug inhibitory interactions were not associated with the severity of intoxications in this study. Cobicistat or ritonavir boosted antiviral regimens are potent inhibitors of drugs metabolism via the cytochrome P450, particularly the 3A4 isoenzyme, resulting in high plasma drug concentrations of ketamine and erectile dysfunction drugs when are coadministrated [18, 19]. Instead, cobicistat or ritonavir boosted antiviral regimens are weak inhibitors of the cytochrome P450 2D6 isoenzyme, and therefore, it is not expected an increasing in plasma concentrations of GHB, mephedrone or methamphetamine [18, 19]. In addition GHB and methamphetamine were the main recreational drugs used, while ketamine and dysfunction erectile drugs were used in a small proportion of patients, which could justify the low percentage of severe intoxications observed in this study.

However, this study revealed that one third of the patients had a possible drug interaction, so clinicians should be aware of the most relevant interactions and switch antiretroviral treatment with lower propensity for drug interactions based on the pharmacokinetics of antiretrovirals and chemsex drug use patterns [18, 19].

The study had some limitations, namely the small number of patients included and lack of a non-PLHIV group due to the low number of non PLHIV admitted to the emergency department for chemsex associated intoxications during the follow-up. Moreover substance use pattern was not evaluated, which could have affected the risk of severe intoxications. However, cases of acute chemsex drug intoxication were well documented by urine drug testing and a long follow-up was employed to evaluate incidence changes over time.

Nevertheless, the study has some implications. The increasing number of chemsex drugs intoxications observed in the study reflects a local deficit in Spanish health responses to chemsex harms. In fact, chemsex care continues to focus on external heroin-based clinics, where men who have sex with men do not attend because they do not feel identified, or community organizations, many of which are not connected to the Spanish health network [42]. As chemsex is interlinked with other sexual and mental health issues, it is essential integrated approaches between different health care services, including HIV/sexual health, psychology, drug and addiction services, to be able to value and offer treatment to chemsex [43].

## Conclusions

In conclusion, acute chemsex drug intoxications in PLHIV are an increasing clinical problem with potential life-threatening events, particularly for whose with low CD4 cell counts and mental health disorders. Chemsex should be routinely screened and properly addressed in the clinical practice, through a multidisciplinary approach, including medical and psychosocial services.

## Data Availability

The datasets generated and/or analysed during the current study are not publicly available due to the fact that we do not wish to share our dataset since they are part of patients’ medical history. However, they are available from the corresponding author on reasonable request.
